# Emerging Roles of MicroRNAs in Diffuse Large B-Cell Lymphoma: From Molecular Mechanisms to Clinical Translation, Liquid Biopsy, and Precision Medicine

**DOI:** 10.3390/biomedicines14091904

**Published:** 2026-08-26

**Authors:** Corina Joldes, Laura Jimbu, Oana Mesaros, Madalina Onciul, Bogdan Fetica, Mihnea Zdrenghea

**Affiliations:** 1 Department of Hematology, Iuliu Hatieganu University of Medicine and Pharmacy, 8 Babes Street, 400012 Cluj-Napoca, Romania; ioana.jimbu@umfcluj.ro (L.J.); mesaros.oana@umfcluj.ro (O.M.); onciul.ioana.madalina@elearn.umfcluj.ro (M.O.); mzdrenghea@umfcluj.ro (M.Z.); 2 Department of Hematology, Ion Chiricuta Oncology Institute, 34-36 Republicii Street, 400015 Cluj-Napoca, Romania; 3 Department of Pathology, Ion Chiricuta Oncology Institute, 34-36 Republicii Street, 400015 Cluj-Napoca, Romania; feticab@yahoo.com

**Keywords:** microRNA, DLBCL, liquid biopsy, biomarkers, prognostic, precision medicine, treatment resistance

## Abstract

Diffuse large B-cell lymphoma (DLBCL), the most prevalent subtype of non-Hodgkin lymphoma (NHL), is an aggressive and fairly heterogeneous group with diverse clinical, pathological, and molecular features. Despite the success of first-line immunochemotherapy, relapsed or treatment-resistant forms remain a clinical challenge. Consequently, minimally invasive markers are needed to predict refractoriness. Recently, circulating microRNAs (miRNAs) have emerged as highly stable, promising biomarkers. MiRNAs such as miR-21, miR-155, miR-222-3p, and the miR-17~92 cluster act as oncogenes that promote tumor survival, while others, such as miR-34, miR-181a, miR-144, miR-101, miR-10a, and miR-320, act as tumor suppressors. Despite multiple recent publications that have correlated dysregulated miRNAs with diagnostic and prognostic importance, the clinical translation has not been fully addressed. Overall, this review provides an updated perspective on the potential of miRNAs in DLBCL and outlines key challenges and future directions for their integration into precision oncology, as miRNAs can remodel the clinical management of DLBCL by enabling earlier diagnosis, improved prognostication, and personalized treatment approaches.

## 1. Introduction

DLBCL is recognised as the most common subtype of NHL. Featuring significant molecular and clinical diversity, it comprises approximately 40% of NHL cases [1]. While DLBCL can develop at any stage in life, it is, like most cancers, a disease of the elderly, with the average age at diagnosis in the mid-60s in Western populations [1,2]. The incidence of DLBCL is rising, with reported increases of 11% in the United States (US) and 7% in Western Europe (WE) in the last 25 years [3]. Risk factors include genetic predispositions, immune system imbalances, virus-related causes, and environmental factors [4]. There is a recognised association between Epstein–Barr virus (EBV) and DLBCL [5]. DLBCL encompasses various molecular groups that reflect the stage of B-cell development from which the disease arises and the activity of distinct biological processes [6]. Based on the cell of origin (COO), the classification distinguishes two main categories: germinal center B-cell-like (GCB) DLBCL and activated B-cell-like (ABC) DLBCL [7]; however, about 15% of cases do not fit either subtype and remain unclassified [6,8]. New classification systems for DLBCL distinguish disease subtypes by integrating genetic, molecular, and immunophenotypic data, leading to a better understanding and more tailored treatment approaches [9]. The standard of treatment in the first line is a combination of rituximab, an anti-CD20 antibody, with prednisone and chemotherapy (cyclophosphamide, doxorubicin, and vincristine) (R-CHOP). About 60% of patients respond with conventional treatment. Cases with relapsed or refractory DLBCL after first-line therapy historically faced a poor prognosis. Those who relapse within 12 months or are primary refractory have particularly poor outcomes, often showing limited response to traditional salvage chemotherapy [10,11]. For a long time, high-dose chemotherapy and autologous stem cell transplantation (HDT-ASCT) were considered curative options for DLBCL cases that were refractory or relapsed. Only around half of these patients qualify for HDT-ASCT, and among those eligible, just 20% achieve a cure. Most patients experience disease progression or complications, making it difficult for them to complete the intensive treatment [12,13]. A major shift in treatment for refractory cases occurred with the introduction of chimeric antigen receptor T-cell (CAR-T) therapy [13]. A variety of therapeutic methods are now available for treating DLBCL. These include several non-molecular biology-based options, such as monoclonal antibodies, antibody–drug conjugates, radioimmunotherapy, CAR-T therapy, and bispecific antibodies. In addition, tailored therapies targeting the B-cell receptor (BCR)/Nuclear Factor kappa-light-chain-enhancer of activated B-cells (NF-κB pathway), B-cell lymphoma 2 (BCL2), exportin-1 (XPO-1), Enhancer of Zeste Homolog 2 (EZH2), and proteasome inhibitors are also being utilized to enhance treatment effectiveness [14].

Despite treatment progress, many patients still experience relapses or have diseases that do not respond to treatment. There continues to be a significant gap in therapeutic options for refractory cases, as well as in the accurate determination of prognosis [14]. To improve the accuracy of prognosis determination, new biomarkers have been explored.

One of the most noteworthy biomarkers is ctDNA. This has gained considerable attention, mainly following its initial observations in solid tumors [15]. It is released into the bloodstream by malignant cells [15]. Research indicates that ctDNA may serve as a valuable non-invasive biomarker for predicting treatment responses and enabling early detection of recurrence in cases of DLBCL [16]. CtDNA was used as a biomarker to assess molecular response in clinical trials evaluating therapeutic agents, including polatuzumab vedotin and odronextamab. Cases that achieved negative ctDNA tests during treatment or by the end of the study protocol often had longer survival [17]. While ctDNA primarily reflects tumor-derived genomic alterations and tumor burden, miRNAs capture information on tumor-associated biological processes, treatment response, and metastatic ability, providing complementary information in liquid biopsy [18].

Recently, there has been increased interest in various other circulating biomarkers, including coding RNAs, such as mRNA, as well as non-coding RNAs, including miRNAs and circular RNA (circRNAs) [19]. MicroRNAs are approximately 18-22-nucleotide non-coding RNAs that regulate gene expression after transcription [20]. They influence nearly every aspect of cancer biology and are increasingly recognised for their roles in different hematological malignancies [21]. In DLBCL, miRNAs affect cell differentiation, growth, programmed cell death, and responses to treatment. Their dysregulation contributes to B-cell malignancies by activating oncogenes or silencing tumor suppressors [22]. Their small size, stability in circulation, and tissue-specific expression profiles make them perfect candidates for diagnostics, prognostics, and therapy [14].

In DLBCL, specific miRNAs have altered expression patterns due to genetic mutations, epigenetic changes, transcriptional deregulation, or oncogenic signalling pathways. All these alterations contribute to tumor initiation and progression, subtype differentiation, drug resistance, and poor prognosis [23]. MiRNAs can serve a dual function in DLBCL, acting either as oncogenes or tumor suppressors by modulating the expression levels of specific related oncogenes. Oncogenic miRNAs, referred to as oncomiRs, promote tumor growth by inhibiting the mRNAs of tumor suppressor genes. Conversely, tumor suppressor miRNAs (TS miRNAs) function by inhibiting oncogene messenger RNAs, leading to their underexpression in cancer [24].

Despite promising evidence of the relevance of miRNAs in DLBCL, the current literature is fragmented across individual miRNAs, molecular pathways, disease subtypes, and clinical use. The interaction between dysregulated miRNAs and signalling pathways involved in DLBCL development, progression, and treatment responses has not been integrated. This review focuses on molecular signalling pathways regulated by miRNAs in DLBCL, integrating their roles as potential diagnostic, prognostic, and therapeutic biomarkers.

Fields where further research may improve the development of more specific and efficient therapeutic strategies need to be identified. A comprehensive and current understanding of the functions of miRNAs is essential to facilitate their effective clinical translation.

## 2. Molecular Signalling Pathways Regulated by miRNAs in DLBCL

**Figure 1:**
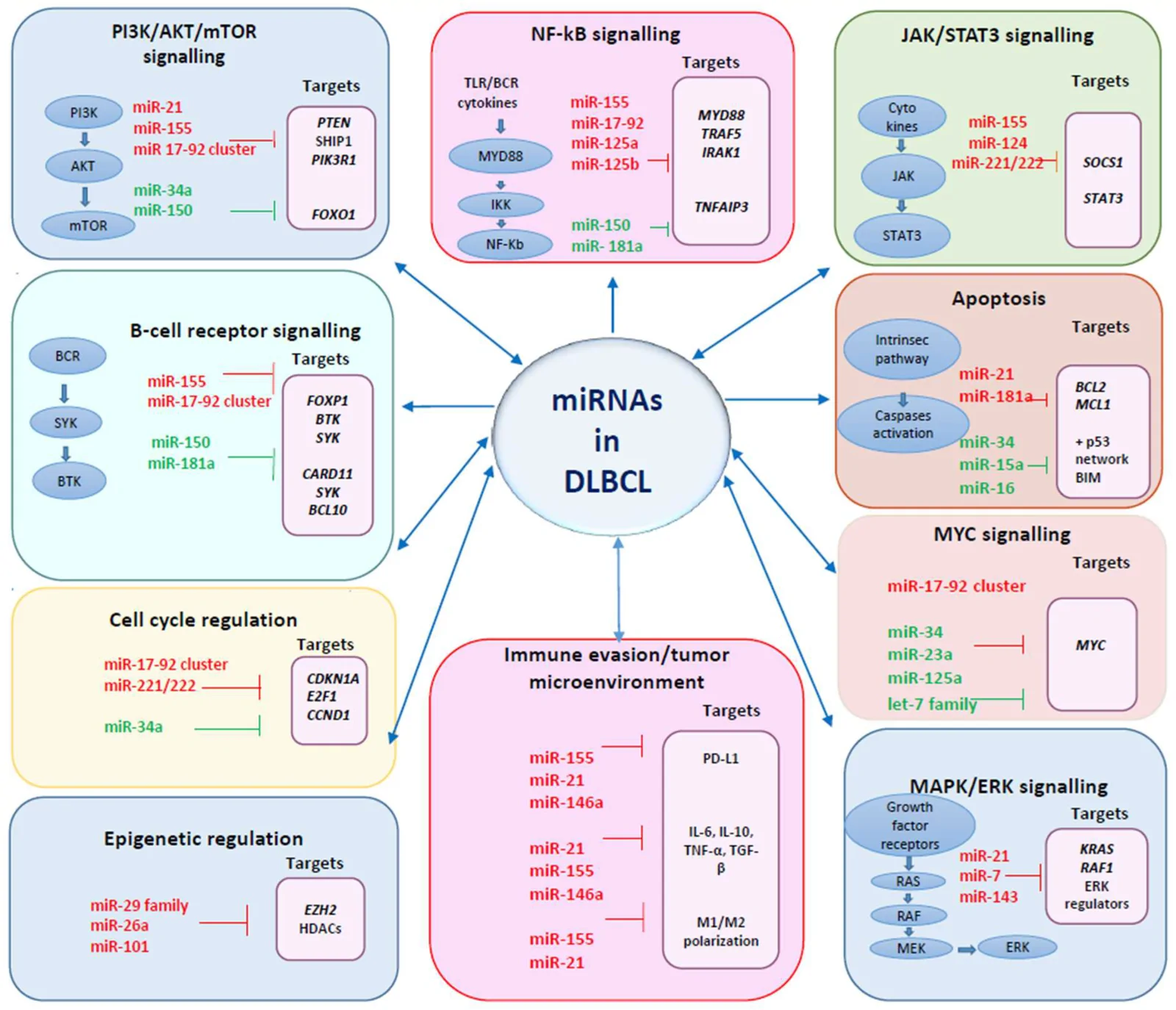
Simplified schematic overview of major signaling pathways regulated by miRNAs in DLBCL. Red miRNAs—oncogenic miRNAs; green miRNAs—tumor-supressive miRNas.

MiRNAs function as key modulators of the signalling network underlying lymphoma development [25], promoting cell proliferation and survival, apoptosis inhibition, supporting angiogenesis, immune responses, and modulating chemosensitivity [23]. The included miRNAs were selected based on their reported functional relevance in DLBCL and evidence of their involvement in regulating key genes involved in oncogenic and tumor-suppressive signalling networks [19,26,27,28,29,30,31,32,33,34,35,36]. The major signalling pathways regulated by miRNAs in DLBCL include BCR signalling, the NF-κB pathway, phosphoinositide 3-kinase/protein kinase B (PI3K/AKT), or MYC-associated regulatory networks, as illustrated in Figure 1.

### 2.1. BCR Signalling

BCR signaling is central to the survival of many DLBCL cells, particularly the ABC subtype [37]. While normal B cells rely on antigen-independent BCR signaling for survival, ABC subtype cells exhibit an aberrant “chronic active” form of BCR signaling. Chronic activation is driven by somatic mutations or autoantigen engagement rather than by external antigen stimulation. This induces spontaneous receptor clustering that continuously increases cell proliferation and prevents apoptosis [38]. Chronically active BCR signaling, mainly in ABC-DLBCL, activates downstream pathways such as NF-κB, the phosphatidylinositol-3-kinase/AKT/mammalian target of rapamycin (PI3K/AKT/mTOR), and MAPK/ERK, leading to lymphomagenesis and therapeutic resistance [39]. The clinical importance of this pathway is underscored by the success of inhibitors targeting the BCR pathway, such as ibrutinib, a Bruton’s tyrosine kinase (BTK) inhibitor, which is highly effective against the ABC-DLBCL subtype [40]. MiRNAs serve as post-transcriptional modulators of this cascade by directly targeting core BCR components [41]. Notably, spleen tyrosine kinase (*SYK*), along with other oncogenes such as Interferon Regulatory Factor 4 (*IRF4*), PIM1 proto-oncogene, serine/threonine kinase (*PIM1*), Forkhead box P1 (*FOXP1*), B-cell Lymphoma 10 (*BCL10*), RUNX family transcriptor factor 1 (*RUNX1*), and ABL proto-oncogene 1, non-receptor tyrosine kinase (*ABL1*), has been identified as a target of miR-129-5p in DLBCL [42]. Consequently, miR-129-5p functions as a tumor suppressor by restraining proliferation and inducing apoptosis in the ABC subtype [43]. In a recent study, miR-370-3p, miR-381-3p, and miR-409-3p have been linked to BCR signaling and MAPK during DLBC progression. Modulating these pathways increases chemosensitivity. MiR-129-5p was downregulated and linked to adverse outcomes [42]. Beyond miR-129-5p, the BCR pathway is tightly coordinated by an opposing network of oncogenic and tumor-suppressive miRNAs [9]. The oncogenic miR-17~92 cluster and miR-155 trigger survival signals by targeting the BCR/NF-κB pathway and PI3K, while tumor-suppressive miRNAs inhibit these pathways. Specifically, miR-150 directly represses *FOXP1* to dampen receptor hyperactivation, while miR-181a downregulates Caspase Recruitment Domain Family Member 11 (*CARD11*), halting downstream pro-survival transcription [9,44].

### 2.2. NF-κB Signalling Pathway

Constitutive activation of the NF-κB pathway is characteristic of the ABC subtype [45], mainly driven by chronic BCR signalling and alterations in genes such as *CARD11*, *CD79B*, or *MYD88* [46]. Sustained NF-κB activation promotes malignant cell proliferation, impairs apoptosis, blocks B-cell differentiation, and accentuates the tumor microenvironment through enhanced inflammation, angiogenesis, and metastasis [47]. Besides these now well-established genetic mechanisms, various miRNAs have been involved in the regulation of the NF-κB signalling pathway. Experimental evidence has shown that miR-125a/b targets lead to increased NF-κB activity and more aggressive DLBCL [48]. Experimental miRNA studies also demonstrate miR-155 or miR-17-92 cluster association with NF-κB activity, promoting malignant cell survival and proliferation [49]. Conversely, miR-150 and miR-181 are known to indirectly hinder this pathway by targeting oncogenic regulators [50,51]. Additional miRNAs involved in NF-κB regulation through targeting of tumor necrosis factor (TNF) receptor-associated factor 5 (*TRAF5*) and other pathway components have been detected by bioinformatics analyses in a recent study [52]. While miR-15a-5p, miR-147a, miR-192-5p, miR-532-5p, and miR-650 are downregulated, miR-197-3p is upregulated, leading to *TRAF5* downregulation and subsequent activation of downstream signaling. In addition to *TRAF5*, this miRNA signature concurrently modulates an extended network of 38 downstream NF-κB pathway components, including *BCL2*, *MYD88*, X-linked inhibitor of apoptosis (*XIAP*), and interleukin-1 receptor-associated kinase 1 (*IRAK1*), either by promoting mRNA degradation or repressing translation [52]. These data should be regarded as hypothesis-generating, providing a framework for future research, as conclusions require validation through experiments and an independent clinical cohort.

### 2.3. PI3K/AKT/mTOR Signalling Pathway

The PI3K/AKT/mTOR axis is an important factor in oncogenesis and therapeutic resistance in 50–70% of cases of DLBCL, regulating cell proliferation, survival, and apoptosis, and is frequently dysregulated in DLBCL [53]. Constitutive activation of this pathway is a key feature of lymphomagenesis. It can happen through multiple mechanisms, such as loss of tumor suppressors like *PTEN*, genetic alterations in PI3K signalling, and chronic activation of BCR signaling [54]. In addition to genetic and receptor-mediated mechanisms, non-coding RNAs have emerged as important modulators of PIK3/AKT/mTOR signalling in tumorigenesis [55]. Several miRNAs have been implicated in the regulation of survival pathways that intersect with PI3K/AKT signalling. MiR-21 is frequently overexpressed in DLBCL and contributes to lymphoma progression by suppressing tumor suppressor genes, including *PTEN*, thereby directly enhancing AKT pathway activity [28]. Similarly, the miR-17-92 cluster functions as an oncogenic regulator in B-cell lymphomas and promotes proliferation and survival through targeting multiple tumor suppressors involved in PIK3/AKT regulation [56]. In addition, miR-155 enhances B-cell proliferation and survival and has been associated with signaling networks involving PIK/AKT pathway activation [57]. In contrast to direct microRNA-mediated regulation, long non-coding (lnc)-associated competing endogenous RNA (ce) networks provide an additional regulatory layer. It has been shown that the lncRNA *ARAPI-AS1* promotes progression in DLBCL by sponging miR-508-5p, resulting in repression of downstream targets such as *EMP1* and activation of PI3K/AKT signaling. *ARAP1-AS1* knockdown hinders proliferation. Pharmacological inhibition of PI3K signaling attenuates its oncogenic effects, supporting the importance of this regulatory axis [58].

### 2.4. Apoptosis and Cell-Cycle Regulation

#### 2.4.1. MYC-Associated Regulatory Networks

The proto-oncogene *MYC* encodes a key transcription factor that promotes the proliferation and survival of aggressive B-cell lymphomas. Dysregulation of the *MYC* family is a proven driver of oncogenesis, therapy resistance, and poor prognosis [14]. *MYC* aberrations are involved in the histological transformation of indolent lymphomas into high-grade lymphoma [59,60]. MYC directly transactivates the miR-17-92 cluster [23,61]. Among miRNAs involved in MYC signaling, miR-34a acts as a tumor-suppressive miRNA by directly targeting *MYC*, inhibiting cell proliferation and promoting apoptosis [62]. Specific tumor-suppressive miRNAs, including miRNA-125a, miRNA-23a, and let-7a, regulate *MYC* expression. The aforementioned miRNAs are downregulated in DLBCL. Disrupting homeostatic feedback loops and upregulating *MYC*, they ultimately sustain tumor cell survival and proliferation [33].

#### 2.4.2. TP53

The tumor suppressor *TP53* regulates cell-cycle arrest, apoptosis, and genomic stability [63]. In DLBCL, *TP53* mutations are associated with aggressive disease, chemoresistance, and poor clinical outcomes, with increased prevalence at relapse [64]. Beyond its canonical transcriptional functions, p53 forms a bidirectional regulatory network with miRNAs, in which p53 controls the expression of several tumor-suppressive miRNAs, while these reinforce p53 activity by targeting its negative regulators [65]. Wild-type p53 transcriptionally activates multiple miRNAs involved in apoptosis and cell-cycle control, amplifying its tumor-suppressive response. Conversely, *TP53* mutations disrupt this regulatory circuit by impairing both the transcription and maturation of p53-responsive miRNAs, resulting in loss of posttranscriptional regulation of oncogenic pathways [66,67]. MiR-34a is a direct transcriptional target of p53. After p53 activation, miR-34a inhibits oncogenic targets, such as *MYC* or *BCL2*, inhibiting proliferation. *TP53* can impair these tumor-suppressive mechanisms by reducing miR-34 expression. MiR-17-92 collaborates with MYC and promotes proliferation, having a more pronounced oncogenic effect when the pathway is impaired [68]. MiR-155 can suppress p53 indirectly by targeting regulators of the p53 pathway, promoting survival and restraining apoptosis. MiR-125b targets *TP53* mRNA directly, reducing protein expression. Overexpression therefore has oncogenic activity by weakening the p53 response. *TP53*, alongside miR-155 and miR-125b, acts as a critical indicator for risk assessment in non-germinal center cases [69]. A considerable set of p53-responsive miRNAs was recently observed [70,71,72]. A decline in the levels of tumor suppressor miRNAs, including miR-34a, miR-34b, miR-34c, miR-129, miR-203, and miR-145 in DLBCL, paralleling those in non-cancerous lymphoid tissue, was noted. These microRNAs are responsive to p53 regulation [73]. Research demonstrated that deregulation of p53-responsive miRNAs in DLBCL is not explained solely by *TP53* mutations. Epigenetic silencing, chromosomal alterations affecting miRNA loci, and defects in miRNA biogenesis cooperate to suppress the p53–miRNA network. The findings highlight that disruption of this axis results from multiple complementary mechanisms that vary among DLBCL subtypes [73].

## 3. Diagnostic and Prognostic Implications of miRNAs in DLBCL

MiRNAs possess several features of ideal biological markers, including stability, conservation across species, and distinct expression patterns corresponding to tissue or clinical stages of the disease [74]. Due to their involvement in the pathogenesis of DLBCL, their diagnostic and prognostic significance, and their roles in treatment response and chemoresistance, numerous miRNAs (Table 1) have been highly studied to facilitate their translation from discovery to clinical application [9].

### 3.1. Oncogenic microRNAs and Prognosis in DLBCL

MiR-21 shows the strongest replication across studies as a non-invasive diagnostic and prognostic marker [75]. MiR-21 plays an important oncogenic role in DLBCL by modulating the PI3K/AKT/mTOR/FOXO1 pathway at multiple levels, with strong prognostic implications [28]. It is involved in the downregulation of phosphatases, including phosphatase and tensin homolog (PTEN) and programmed cell death protein 4 (PDCD4). These phosphatases are vital in the PI3K/AKT and mitogen-activated protein kinase (MAPK) signaling pathways [75]. For miR-21, the literature is conflicting. Some studies show elevated miR-21 expression associated with poor overall survival (OS) or progression-free survival (PFS), advanced stages, and chemoresistance [28,85]. Increased expression of miR-21 predicts prognosis in DLBCL. In a cohort of 112 DLBCL cases, serum levels were significantly increased and correlated with B constitutional symptoms and the IPI score. Thus, high miR-21 is considered a predictor of worse overall survival [85]. Another study using tissue samples demonstrates the prognostic implications of miR-21 in DLBCL. Overexpression was associated with shorter PFS and OS in cases treated with rituximab-containing chemotherapy [28]. Conversely, other studies did not identify a significant association between miR-21 expression and prognosis in a series of 56 tissue samples from DLBCL cases [86]. Other suggested that elevated miR-21 levels may be linked to a favorable response in specific therapeutic regimens. Elevated miR-21 expression was associated with improved relapse-free survival, but no correlation with clinical or pathological variables was observed [78]. Higher serum miR-21 levels in DLBCL patients were associated with favorable relapse-free survival and poor OS in tissue samples in 18 studies, including 1950 patients with DLBCL eligible for pooled analysis [87]. These inconsistencies among studies are due to heterogeneity in study design, with different cohort sizes, treatment regimens, or biological material analyzed. They should be taken into account when interpreting the clinical utility of miR-21 [9].

Dysregulation of miR-155 expression is linked to various hematologic and solid tumors, making it a significant oncomiR in cancer research [26]. The oncogenic characteristics of miR-155 are illustrated in Eμ-miR-155 transgenic mice, which developed aggressive pre-B-cell neoplasms within 3 to 4 weeks [26,88]. In aggressive B-cell lymphoma, it is one of the most constantly overexpressed miRNA [26]. One foundational study showed that DLBCL cells have higher levels of miR-155 than normal circulating B cells [89]. Serum miR-155 levels were significantly increased in newly diagnosed DLBCL patients, strongly correlated with tumor tissue expression, and predicted poor disease outcome by modulating the tumor microenvironment [90,91]. MiR-155 expression in extracellular vesicles is significantly elevated in relapsed and refractory DLBCL cases compared to those responding to treatment [92,93]. As it is the best-characterized oncomiR in DLBCL, it can serve as a perfect bridge between molecular biology and clinical application [76]. Gene expression profiling studies have shown elevated levels of miR-155 in DLBCL compared to reactive lymph tissue, and notably higher levels in the ABC subtype compared to GCB-DLBCL [84]. This suggests a possible use in molecular classification. Because the ABC subtype has a more aggressive clinical course and an inferior response to immunochemotherapy, miR-155 expression may serve as a complementary diagnostic marker alongside molecular classification [77,94]. The potential of miR-155 as a prognostic biomarker in cases of DLBCL is under discussion. Multiple studies have linked miR-155 expression to adverse clinicopathological traits, including higher International Prognostic Index (IPI) scores, advanced disease stages, extranodal disease, inferior OS and PFS, especially in the ABC subtype, or therapy resistance [84,95]. Some studies failed to identify miR-155 as an independent prognostic factor in DLBCL [96,97,98]. Further prospective multicenter studies using standardized methods are needed before implementing miR-155 as a prognostic biomarker [26].

The miR-17~92 cluster (miR-17, -18a, -19a, -19b-1, -20a, and -92a-1) is overexpressed in DLBCL. Studies using DLBCL-induced mouse models have sustained its oncogenic potential, indicating that elevated miR-17~92 expression contributes to dysregulation of cell proliferation and survival pathways involved in DLBCL [99]. Functional genomic studies indicate that the miR-17~92 cluster can activate MYC, thereby significantly contributing to DLBCL progression by silencing key tumor suppressor genes [61], such as *PTEN* and *BIM* (*BCL2L11*), which are essential for apoptosis [100]. The regulatory pathway involving the miR-17~92 cluster is critically associated with MYC, contributing to the miR-17-92/MYC/E2F circuit [101]. This circuit facilitates the upregulation of the miR-17-92 cluster and targets E2F1, an important regulator of cell proliferation. Additionally, the *E2F3* gene plays a supportive role alongside E2F1 in promoting the expression of the miR-17-92 cluster [102]. The miR-17~92 cluster is a critical driver of DLBCL, serving as a reliable diagnostic tool to differentiate GCB-DLBCL from FL and as a significant prognostic biomarker associated with poor survival [103].

MiR-222-3p has been found to play an oncogenic role in DLBCL [22]. Broadly, miR-222-3p exhibits context-dependent functions in human cancers and has been associated with multiple processes involved in tumor development and progression [95]. In DLBCL, miR-222 expression acts as an independent predictor of outcome, with higher levels associated with shorter PFS [96]. Recently, it was proposed that the lymphocyte-to-monocyte ratio (LMR), which is significantly associated with survival in DLBCL cases, correlates with serum levels of miR-222-3p. This may reflect important immunological interactions and could serve as a non-invasive prognostic biomarker [97].

### 3.2. Tumor-Suppressive miRNAs and Prognosis in DLBCL

Among the tumor-suppressive miRNAs explored in DLBCL, miR-181a, miR-34, miR-150, or the miR-29 family have appeared as potential regulators of lymphoma progression, despite differing clinical evidence among them [83,104,105,106].

MiR-181a is a tumor-suppressive microRNA and serves as a prognostic marker in DLBCL [107]. Higher levels of miR-181a expression are associated with better clinical outcomes. This microRNA acts as a negative regulator of the NF-κB signaling pathway. It is usually downregulated in the more aggressive ABC subtype of DLBCL when compared to the GCB subtype. The *CARD11* gene was identified as a potential target of miR-181a [107]. MiR-34a, an element of the p53 regulatory network, is repeatedly deregulated in DLBCL. Reduced expression is linked to aggressive forms of disease and chemoresistance [82]. Decreased miR-34a levels in tissue samples of DLBCL and MALT lymphoma, coexisting with increased FOXP1, p53, and BCL2 protein, predict poor OS and can represent prognostic markers in clinical practice [83]. Other tumor suppressors, such as the miR-29 family or miR-150, have antiproliferative and antiapoptotic effects, but their prognostic value needs further validation [108]. The prognostic value of miR-150 in primary gastrointestinal (PGI)-DLBCL was assessed by assessing the association between miR-150 expression and clinicopathological characteristics in patients with PGI-DLBCL. Lower miR-150 levels were observed in tissue samples, and miR-150 expression was significantly lower in patients with high IPI scores than in patients with low IPI scores. Downregulated miR-150 expression was significantly associated with shorter OS and PFS [109]. In DLBCL, the miR-29 family (including miR-29a, miR-29b, and miR-29c) generally works as a tumor suppressor. In aggressive B-cell lymphomas, miR-29 is frequently downregulated, often driven by oncogenic drivers like *MYC*. This disturbs essential controls on cell growth and survival pathways, such as the PI3K/AKT signaling pathway, resulting in rapid proliferation and treatment resistance [34]. MiR-497 and miR-199a have arisen as promising tumor-suppressive miRNAs in DLBCL. High expression was associated with better OS. Their overexpression also modified drug sensitivity in DLBCL cells, suggesting a new therapeutic target for DLBCL [106]. Because of the heterogeneous prognostic potential of tumor suppressors in DLBCL, integrating complementary candidates rather than individual miRNAs could enhance their prognostic value, offering a new approach to precision medicine in DLBCL [110].

### 3.3. Other miRNAs Correlated with Outcomes in DLBCL

The overexpression of miR-645 is linked to cell proliferation and programmed cell death. MiR-645 targets the *DACH1* gene by binding to the 3’ UTR of the *DACH1* mRNA transcript. This leads to a decrease in *DACH1* expression in DLBCL cells [111]. This reduced expression of DACH1 is associated with increased cell proliferation and enhanced cell survival. *DACH1* is implicated in various malignancies and is correlated with poor outcomes and tumor progression [23].

In DLBCL, high serum miR-22 expression was associated with poorer prognosis [112]. Higher serum miR-22 levels at diagnosis are associated with a poorer PFS in patients. Those with elevated miR-22 levels are at a greater risk of developing a resistant form of the disease or experiencing a relapse after standard therapy. This association remains significant regardless of the IPI score. Therefore, serum miR-22 could serve as a non-invasive prognostic biomarker to help stratify newly diagnosed patients with DLBCL [112].

MiR-665 is downregulated in the serum samples of patients with DLBCL, according to miRNA sequencing [113]. To investigate the regulation and the mechanism of miR-665 in the progression of DLBCL, a bioinformatics analysis was conducted to predict potential target genes of miR-665. It was noted that LIM and SH3 protein 1 (*LASP1*) is a probable target of miR-665 [113]. LASP1 is a protein first identified from the cDNA of a breast cancer patient with metastatic axillary lymph nodes [114]. In DLBCL, miR-665 acts as a tumor-suppressive microRNA by down-regulating *LASP1* and *MYC*. This suppression may reduce lymphoma cell proliferation and malignant invasion, and possibly increase apoptosis or decrease survival signalling. Therefore, miR-665 might serve as both a prognostic biomarker and a potential therapeutic target in DLBCL cases [115].

Primary testicular (PT) DLBCL is an isolated aggressive lymphoma with different clinical and molecular features [116]. PT DLBCL is related to a high risk of relapse or recurrence mainly in the “immune sanctuary” sites, such as the central nervous system and contralateral testis, showing inferior clinical outcomes [117]. A recent study on a large cohort attempted to identify prognostic biomarkers in PT DLBCL cases by evaluating RNA and DNA specimens [118]. Examination of genomic microRNA profiling has revealed a specific microRNA signature with substantial differential expression between patients with PT DLBCL and those with systemic DLBCL. Moreover, in the PT DLBCL cohort, patients showing higher levels of 16 characteristic microRNAs (miR-514a-3p, miR-202-3p, miR-509-5p, miR-509-3-5p, miR-506-3p, miR-508-3p, miR-514b-5p, miR-513a-5p, miR-510-5p, miR-513b-5p, miR-513c-5p, miR-508-5p, miR-509-3p, miR-202-5p, miR-507, and miR-514b-3p) revealed higher survival outcomes [118].

## 4. Liquid Biopsies in DLBCL

To enhance the evaluation of prognosis and treatment response in DLBCL, new biomarkers have been investigated. Invasive diagnostic approaches are being progressively substituted by non-invasive methods, such as liquid biopsies [119]. The evaluation of circulating malignant cells can provide tumor-related information. This evaluation is less pertinent in DLBCL, as this type is characterized by the absence of circulating tumor cells [26].

Currently, the examination of circulating tumor DNA (ctDNA) shows the strongest clinical evidence. It represents tumor-derived cell-free DNA and, in DLBCL, reflects the mutational profile of the tissue biopsy, but it can also detect mutations absent from the tissue biopsy. In DLBCL, in addition to genotyping, it can assess tumor burden, early response, minimal residual disease (MRD), or relapse [120,121]. Changes in ctDNA after chemotherapy can anticipate patient outcomes [122]. CtDNA reduction at two cycles of R-CHOP and its combination with interim PET results improve patient prognosis stratification [123]. There are some limitations related to the low tumor burden or lack of assay standardization [124]. Some clinical trials trying to integrate ctDNA into the treatment approach in hematological malignancies are now underway [122]. Compared to ctDNA, circulating miRNAs represent free-cell miRNAs, present in serum or plasma of DLBCL cases. They are promising biomarkers for diagnosis, prognosis, and response assessment, as they are stable and relatively inexpensive [19]. There are some limitations for their use in clinical practice related to the biological heterogeneity [125,126].

Extracellular vesicles (EVs) are nanoscale vesicles released into the extracellular space by nearly every cell type [127]. Due to their presence in various biofluids, including blood, and because of their stability, EVs are promising noninvasive biomarkers [128]. DLBCL-derived EVs transport biomolecules that can promote tumor progression by enhancing cell proliferation, invasion, and resistance to chemotherapy, while also reducing apoptosis [129,130,131,132,133]. In DLBCL cases, EVs derived from malignant cells have been shown to acquire chemoresistance through various mechanisms. MiRNAs carried by EVs take part in posttranscriptional regulation [128]. Exosomes reduce CD20 expression in lymphoma cells by shuttling microRNA-125b-5p, thereby diminishing the effectiveness of rituximab [129]. Other mechanisms by which EVs evade rituximab include the absorption of rituximab and the consumption of complement [134]. DLBCL-cell-derived EVs can adopt the M2 phenotype by regulating the expression of the protein PGC-1β, thereby promoting tumor progression [132]. EVs contain miRNAs that can be delivered to macrophages, regulating the expression of various genes. One such miRNA is miR-7e-5p [128]. A study found that miR-7e-5p was downregulated in high-grade FL and DLBCL compared to indolent cases [135]. Reduced expression of miR-107 and elevated levels of miR-99a-5p, miR-125b-5p, miR-155, CA1, CD-9-CD63, and PD-L1-CD63 are associated with poor prognosis in DLBCL. Variations in the expression levels of Let7g [92], and miR-451 can be considered indicators of therapeutic responses [128].

Recently, the potential of exosomal miRNA profiles as biomarkers has gained attention, particularly for cancer diagnosis and classification [136]. Exosomes are now recognized as information-carrying vesicles, with a crucial role in intercellular communication [137]. Clinical translation is restrained by the absence of standardized isolation methods [138], processing methods, and storage [137]. Isolation still faces three major challenges represented by the absence of standardized miRNA extraction protocols, insufficient mechanistic studies, and technical barriers to translation [137]. Analytical techniques need optimization for sensitivity, specificity, standardization, and validation across multiple centers [139]. Standardized protocols and prospective multicenter validation are essential before clinical application [137]. A recent study investigated the miRNA content of plasma-derived exosomes in DLBCL cases compared with healthy controls. It also examined whether DLBCL-derived exosomes can affect miRNA expression in normal B-cells in vitro [140]. The main findings indicate that plasma exosome concentration is significantly higher in DLBCL cases than in healthy individuals. Intriguingly, in cases with nodal involvement and at more advanced stages, the exosomal concentration is reduced. Using miRNA array profiling, researchers identified 33 miRNAs in plasma exosomes from DLBCL cases that were significantly reduced compared to those from healthy cases. Among these, three miRNAs were emphasized as possible markers for DLBCL diagnosis: miR-3960, miR-6089, and miR-939-5p. It was observed that normal B-cells internalized DLBCL exosomes, and their miRNA expression profiles changed based on the lymphoma subtype from which the exosomes originated. It was also noted that inhibiting the exosome/endosome pathways using bafilomycin A augmented the sensitivity of DLBCL cells to R-CHOP treatment [140].

Diagnostic markers, including miR-19, miR-21, and miR-92a, show promise in differentiating central nervous system (CNS) lymphoma [141]. The precise diagnosis of central nervous system (CNS) involvement in DLBCL is a complex process that is crucial for effective treatment [142]. Cytomorphology, flow cytometry, and biochemical analysis of cerebrospinal fluid (CSF) are crucial for initial screening. As CSF cells decay rapidly, prompt sample handling is required [141]. MiRNAs offer advantages as diagnostic markers due to their stable detection in CSF. This stability stems from the fact that miRNAs are encapsulated in microvesicles, allowing them to maintain their integrity over extended periods and ensure their preservation [143].

CNS relapse occurs in about 5% of patients with DLBCL and has a high fatality rate [144]. Identifying molecular markers for CNS involvement is crucial for accurately stratifying patients’ risk for relapse. MiRNAs were analyzed from patients with newly diagnosed DLBCL who had clinical risk factors for CNS involvement. Two differentially expressed miRNAs, miR-20a and miR-30d, that predict CNS involvement were identified. The results suggest that these miRNAs could help refine the risk stratification for patients at risk for CNS relapse in DLBCL. Further large-scale studies are needed to elucidate the underlying pathways [145].

## 5. Subtype-Specific miRNA Signatures in DLBCL

**Figure 2:**
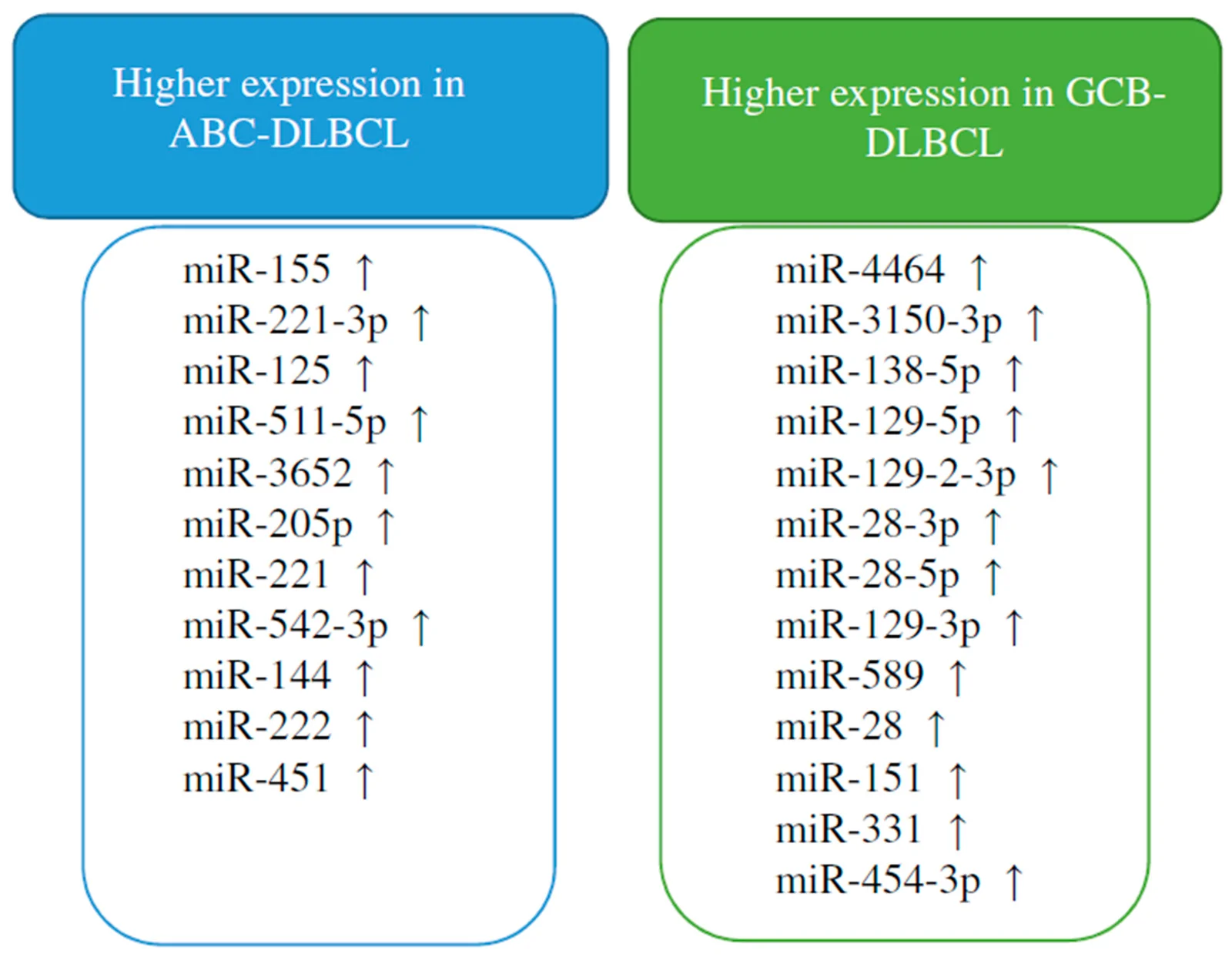
Differentially expressed miRNAs in ABC-DLBCL and GCB-DLBCL. Arrow—upregulated miRNAs in the indicated DLBCL subtype.

Differentially expressed miRNAs have been studied to distinguish the two COO tumor subtypes. The GCB-DLBCL and ABC-DLBCL subtypes are differentiated by their distinct miRNA profiles (Figure 2). [23,77,146,147,148].

A systematic review using tissue samples demonstrated that ABC-DLBCL exhibits upregulated levels of miR-155 and miR-221-3p. These miRNAs suppress *PIK3R1* and consequently activate the PI3K/AKT signaling pathway [74]. In cases of non-GC DLBCL, a link between the expression of miR-125b and miR-155 and disease progression and prognosis was shown using tissue samples [143]. Additionally, *TP53* and *MYC* are associated with these miRNAs and may act as reliable biomarkers for evaluating prognosis in DLBCL. However, more data from a larger cohort is needed to confirm these findings [14,143]. A next-generation sequencing (NGS) study of formalin-fixed paraffin-embedded (FFPE) samples from ABC and GC cases described differences in miRNA expression among DLBCL subtypes [149]. In the non-GC subtype, the following microRNAs were identified as upregulated: miR-511-5p, miR-3652, and miR-205-5p. In the GC subtype, the microRNAs that exhibited upregulation include miR-129-2-3p, miR-4464, miR-3150-3p, miR-138-5p, and miR-129-5p [149].

In mature B-cell lymphomas, in order to distinguish between the GCB and ABC subtypes, a cluster of miRNAs supported differentiation between lymphoma types, such as DLBCL and FL. A group of miRNAs exhibited a coherent expression pattern in aggressive B-cell lymphomas, suggesting the potential to differentiate these malignancies based on miRNA profiling [150]. High-grade lymphoma with 11q aberration (HGBCL-11q) is an aggressive form of B-cell lymphoma that has molecular and histological features of both Burkitt lymphoma (BL) and DLBCL [151]. It is regarded as a particularly aggressive B-cell lymphoma entity in the current WHO classification (WHO-HAEMS5) and typically exhibits a GCB expression signature [150]. MiRNA configurations in HGBCL-11q were not characterized until a recent study, in which the authors sought to define the miRNA expression profile of HGBCL-11q and compare it with BL and DLBCL to determine whether the miRNA profile supports HGBCL-11q as a distinct entity [152]. Unequal gene expression analyses identified several clusters of overexpressed miRNAs in HGBCL-11q compared to BL and DLBCL. The miRNAs that were overexpressed when compared to BL included miR-9-3p, miR-9-5p, miR-3919, miR-129-1-3p, miR-129-2-3p, miR-331-3p, miR-196b-5p, miR-28-5p, and for DLBCL miR-3919, miR-1290, miR-4538, and miR-4791. In particular, miR-3919 showed higher expression levels in HGBCL-11q than in both BL and DLBCL. Additional investigations are required to better understand the role of miR-3919. Its presence in FFPE samples may provide a promising biomarker for the diagnosis of HGBCL-11q [152].

## 6. miRNAs and Therapeutic Targets in DLBCL

Studies indicate that miR-144 functions as a tumor suppressor by inhibiting B-cell lymphoma 6 (BCL6) activity in DLBCL, both in vitro (OCI-Ly3 cells) and in vivo xenograft mouse models [153]. It regulates cell proliferation, apoptosis, and migration, and its low expression is associated with aggressive disease. Increasing miR-144 levels may serve as a therapeutic strategy for DLBCL, given its role as a tumor suppressor [154].

MiR-101 decreases tumor proliferation and progression [155]. The mediated suppression by miR-101 suggests its influence in modulating both apoptosis and cell proliferation via the MAPK/ERK signaling pathway [156]. Findings show that miR-101-5p directly affects *CD47* expression by interacting with its mRNA, possibly leading to its breakdown. In DLBCL, higher levels of miR-101-5p may increase lymphoma cells’ vulnerability to chemoimmunotherapy by reducing CD47 levels on tumor cells. This could improve OS and PFS in patients receiving treatment. These aspects can be used to predict patient outcomes, identifying DLBCL patients with low miR-101-5p who may not benefit from standard chemoimmunotherapy but could gain more from anti-CD47 treatments. Additionally, understanding how CD47 is regulated is essential for improving anti-CD47 therapies. Increasing negative regulators, such as miR-101-5p, or blocking positive regulators, such as MYC, can enhance the immune response against tumors and work synergistically with CD47-blocking antibodies. Identifying other factors that affect phagocytosis could also improve treatment effectiveness. Overall, better knowledge of CD47 could enhance current and future therapies [157].

MiR-10a and miR-187 function as tumor suppressors by regulating *BCL6* expression through induction of apoptosis, as evidenced by cell culture and tumor microarray studies. This could lead to a new therapeutic approach for DLBCL treatment [23].

MiR-320d has a tumor-suppressing function [158]. It acts by downregulating the *CDK6* mRNA transcript at the 3’UTR, leading to inhibition of cell proliferation and cell-cycle arrest. Given this role, it can be considered a potential candidate for therapeutic intervention [159].

MiR-26a is involved in regulating CDK5 activity and p53 expression. This highlights the importance of cell survival, proliferation, and cell cycle progression. Given its influence on CDK5 and p53, miR-26a can be considered in the development of effective treatment strategies for DLBCL [160].

MiR-24 may serve as a potential target in EBV-associated B-cell lymphomas [161]. DLBCL has an inferior prognosis due to its association with EBV. Previous studies have demonstrated that EBV-encoded nuclear antigen (EBNA2) upregulates programmed cell death ligand 1 in DLBCL through downregulation of miRNA-34a [162]. A recent study aims to explore if EBNA2 affects the inducible costimulator (ICOS) ligand (ICOSL) [161]. It was found that in B-cell lymphoma cell lines contaminated with EBV and expressing EBNA2, or in B-cells engineered to express EBNA2, ICOSL expression, protein, and miRNA are considerably reduced compared to EBNA2-negative controls. Research showed that EBNA2 causes high expression of miR-24 (miR-24-3p) in the B-cell lymphoma lines. EBV, through EBNA2, evades host immune surveillance by down-regulating ICOSL via the induction of miR-24. This mechanism reduces the cell’s ability to stimulate T cells via the ICOS/ICOSL axis. EBNA2 utilizes miR-24 not only to evade immune detection, but also to regulate the expression of c-MYC. By increasing the levels of miR-24, c-MYC is maintained at a level that supports cell proliferation while preventing the apoptosis that often results from elevated MYC activity. Inhibiting miR-24 might restore ICOSL expression and tumor immunogenicity, shifting the balance toward apoptosis in tumor cells [161].

MiR-3940-5p has been identified as a regulator that downregulates the expression of LIM domain protein (PDLIM1). The authors proposed that restoring miR-3940-5p or inhibiting PDLIM1 could serve as a therapeutic approach for DLBCL [163]. The *PDLIM1* is a protein-coding gene with selective expression across diverse human malignancies, including DLBCL [164]. The deregulated state of *PDLIM1* was associated with the expansion, spreading, and survival of cancerous cells [163]. A recent study found that *PDLIM1* was overexpressed in DLBCL cases. Higher PDLIM1 levels were associated with poorer OS and disease-free survival (DFS), whereas *PDLIM1* downregulation was associated with improved OS and DFS [163]. In vitro analyses revealed that higher *PDLIM1* levels are associated with increased DLBCL cell proliferation, while *PDLIM1* knockdown reduces proliferation and increases apoptosis [163].

## 7. miRNAs and Treatment Responses in DLBCL

The role of miR-22-3p in regulating ferroptosis in DLBCL via the SIRT1/P53 pathway was explored in a recent study [165]. Ferroptosis is an iron-dependent mechanism of cell death with considerable potential for the development of cancer therapies [166]. Increasing miR-22-3p levels in DLBCL cells lowers SIRT1 levels. This reduction enhances p53 activity, which in turn increases lymphoma cells’ sensitivity to ferroptosis-induced cell death. This research identifies the previously unexplored miR-22-3p/SIRT1/p53 pathway as a regulator of ferroptotic cell death in DLBCL, suggesting it as a potential therapeutic target for this disease [165]. Accelerated cell metabolism and proliferation can disrupt iron homeostasis in hematological malignancies. This can lead to accumulation of redox-active molecules. The tumor microenvironment makes tumor cells particularly vulnerable to ferroptosis [166,167,168,169].

MiR-421 and miR-324-5p have distinct circulating patterns in R-CHOP-refractory and responding DLBCL cases. Small RNA sequencing was utilized to analyze serum samples from 33 DLBCL patients. In the identified miRNAs, expression levels were linked to PFS. MiR-421 and miR-324-5p were differentially expressed in tumor tissues in relation to treatment response. MiR-421 and miR-324-5p were significantly downregulated in tumor tissues of refractory patients compared to responsive ones. Upregulating these miRNAs reduced cell proliferation and resistance to R-CHOP in the GCB subtype. *EGLN1* and *TXNRD1*, which regulate oxygen metabolism and redox homeostasis, were identified as miRNA targets, and their suppression or inhibition impaired cell viability and induced ferroptosis [110].

Overexpression of miR-497 and miR-199a is associated with sensitivity to chemotherapeutic agents such as doxorubicin, rituximab, and vincristine. Furthermore, sensitivity to doxorubicin and rituximab has also been linked to the overexpression of miR-409-3p, miR-370-3p, and miR-381-3p [29]. A review by Alsaadi et al. examined the roles of miR-125b and miR-130, alongside miR-497 and miR-199a, in chemoresistance in DLBCL [106].

## 8. Therapeutic Potential

Modulation of miRNAs offers a possibility to overcome treatment resistance in DLBCL. There are different strategies: inhibiting oncogenic miRNAs using antisense oligonucleotides and miRNA sponges, or restoring tumor-suppressive miRNAs using miRNA mimics and small molecules to intensify the functions of endogenous miRNAs [23,170].

In DLBCL, the most advanced evidence covers miR-155 and miR-21, while miR-17-92 and miR-34a have mechanistic and preclinical support [9,75,94].

MiR-155 is one of the most extensively studied therapeutic targets. The therapeutic relevance is supported by studies showing that pharmacological inhibition of miR-155 can suppress lymphomagenesis [26,98]. A translational study evaluated cobomarsen, designated MRG-106, a locked nucleic acid (LNA) antisense oligonucleotide specifically designed to inhibit miR-155 [76]. Cobomarsen was tested in vitro in DLBCL cell lines. Decreased cell proliferation and increased programmed cell death were noted in DLBCL cell lines with high miR-155. To assess the efficacy of cobomarsen in vivo, a xenografted mouse model harboring DLBCL tumors was used, resulting in a reduced tumor volume. Also, preliminary data were reported for a patient with aggressive ABC-DLBCL who had five cycles of cobomarsen. It was well tolerated, and the tumor burden was reduced, without reported treatment toxicity, suggesting cobomarsen as a potential therapeutic approach in DLBCL [76]. The effects of miR-155 inhibitors are important to enhance treatment response. It was demonstrated that miR-155 depresses *DEPTOR* and *c-CBL*, regulators of BCR downstream signalling. Inhibition of miR-155 reduced NF-kB expression target genes and enhanced sensitivity to the BTK inhibitor, ibrutinib. Lower DEPTOR expression was seen in the more aggressive ABC-DLBCL, suggesting a contribution to biological differences between DLBCL subtypes and potentially different responses to targeted therapy [171].

MiR-21 is another potentially actionable oncomiR in DLBCL. Studies have shown that inhibition of miR-21 with antisense oligonucleotides reduced proliferation and increased apoptosis [172,173]. Inhibition of miR-21 increased protein levels of PTEN and PDCD4, but not corresponding mRNA levels, consistent with post-transcriptional regulation [173]. The relevance of this pathway is strengthened by linking miR-21 to chemotherapy response. In cell lines, an antisense-mediated miR-21 inhibitor augmented the cytotoxic effects of the CHOP regimen. *PTEN* is a target of miR-21, and modulating miR-21 affected the PI3K/AKT signaling pathway, providing a molecular explanation for enhanced sensitivity to chemotherapy. This suggested that miR-21 can modulate treatment responses [174].

The therapeutic modulation of miRNAs is not limited to inhibition of oncomiRs. An alternative is miRNA replacement therapy, restoring miRNAs that are suppressed or lost during lymphomagenesis. Nucleic acids are commonly used, including synthetic miRNAs (miRNA mimics), recombinant expression vectors carrying miRNA-encoding sequences, and oligonucleotide-based miRNA inhibitors (anti-miRs) [175]. Systemic delivery of miR-34a in a xenograft DLBCL model inhibited lymphomagenesis in vivo and induced programmed cell death [174]. Because miRNAs regulate multiple transcripts, restoration of a tumor suppressive miRNA can have side effects in normal tissues. The challenges are related to efficient delivery to malignant cells and avoidance of systemic toxicity [48].

Despite current evidence of the role of miRNAs in the pathogenesis of DLBCL, as well as in molecular classification, diagnosis, prognosis, and treatment response, several limitations restrict their translation into clinical practice as biomarkers of therapeutic targets. One major aspect is the small size of cohorts. This affect statistical significance, with false-positive results and validation failure when replicated in large clinical trials [176]. The heterogeneity of DLBCL is another aspect. The disease encompasses different molecular subtypes characterized by distinct genetic drivers and clinical outcomes. The variability in COO classification, alongside disease stage and status (newly diagnosed/relapsed), or prior treatments can influence miRNA expression patterns, contributing to discordant findings between studies [176]. The type of biological material analysed varies among studies; some include fresh-frozen or formalin-fixed tissue, while others include serum, plasma or extracellular vesicles, with different extraction and storage methods. As storage conditions influence some miRNAs, for accurate quantification the storage methods and biological material should be maintained constant within cohorts [177]. The lack of a universal normalization strategy for circulating miRNA quantification is a source of inconsistency among studies [178]. Additional discordance emerges from different analytical platforms [179]. Further research should prioritize large-scale, multi-center studies that reflect diverse populations, rigorous clinical annotation, standardized pre-analytical and analytical protocols, and integrate multi-omics layers (genomic, cfDNA methylation/fragmentomics, transcriptomics, proteomics, metabolomics) to establish the clinical utility of miRNAs and determine their added value beyond current diagnostic standards [179,180].

An emerging alternative to oligonucleotide-based therapeutics is small-molecule modulators [181]. They target miRNA biogenesis or function by binding to the miRNA precursor. The small molecule AC1MMYR2 (NSC211332) inhibits Dicer-mediated processing of pre-miR-21 [182]. NSC141562 suppresses the *MIR155HG*/miR-155 axis to reduce oncogenic microRNA, providing evidence that oncogenic miRNAs can be targeted [183]. Compared to traditional oligonucleotides, they have better cell permeability and potential for oral administration, but require enhanced selectivity to avoid off-target RNA-binding and toxicity. These strategies are still preclinical and need further validation, but this approach has the potential to reshape cancer treatment [181].

## 9. Conclusions

Even though the DLBCL treatment landscape is evolving, resistant disease or relapse remains a clinical challenge in DLBCL. New biomarkers, particularly miRNAs, offer promising approaches to improve diagnostic and prognostic accuracy, as well as therapeutic strategies. But their clinical significance is not always consistent across studies. Differences in patient cohorts, DLBCL subtypes, treatment regimens, biological specimens, analytical platforms, and normalization methods are some unresolved challenges that contribute to the observed discrepancies. Thus, individual miRNAs should be validated in large, prospective, multicenter cohorts before being considered definitive clinical biomarkers.

For future research, rather than focusing on isolated miRNAs, attention should be given to combinations of miRNA profiles and treatment response signatures. Integration of miRNAs with multi-omics, potentially supported by bioinformatics and artificial intelligence, may contribute to developing multi-marker models for precision medicine.

Overall, miRNAs are a promising bridge between the molecular biology of DLBCL and clinical management.

## Figures and Tables

**Table 1:** Clinical Relevance of miRNAs in DLBCL.

miRNAs and Expression Pattern	Diagnostic Value	Prognostic Value	Clinical Relevance
miR-155 ↑ Upregulated [33,75,76,77,78]	One of the best validated circulating biomarkers	High expression associated with inferior OS/PFS	Linked with aggressive ABC subtype R-CHOP resistance Potential therapeutic target
miR-21 ↑ Upregulated [75,78,79]	Significant value	High expression predicts worse OS/PFS	Predictor of R-CHOP response; inhibition increases chemosensitivity Candidate for liquid biomarker
miR-17-92 cluster (miR-17, miR-18a, miR-19a/b, miR-20, miR-92a) ↑ Upregulated [33]	Subtype differentiation	Associated with MYC-driven aggressive disease	Therapeutic target under investigation
miR-224-5p ↑ Upregulated [80]	Unknown	Unknown	Higher expressions with favourable R-CHOP response
miR-125b ↑ Upregulated [81]	limited	Adverse biology	Posible drug resistance
miR-182-5p ↑ Upregulated [82]	Emerging	Associated with aggressive phenotype	Potential therapeutic target through PI3K signaling
miR-34a ↓ Downregulated [83]	Moderate value	Low expression associated with poor prognosis	Low levels associated with chemoresistance Restoration improves R-CHOP sensitivity Therapeutic mimic investigated (MRX34)
miR-29c ↓ Downregulated [75]	Emerging	Lower levels associated with worse outcomes	Candidate for circulating biomarker
miR-27a/27b ↓ Downregulated [23]	Limited	Low levels associated with poor prognosis	Higher levels associated with R-CHOP response
miR-146a ↓ Downregulated [84]	Limited	Higher expression associated with better prognosis	Increased after chemotherapy and associated with better prognosis

## Data Availability

No new data were created or analyzed in this study. Data sharing is not applicable to this article.
